# Influence of antiplatelet drugs on gastric ulcer healing after endoscopic submucosal dissection in patients with early gastric cancer

**DOI:** 10.1002/deo2.70070

**Published:** 2025-02-11

**Authors:** Kazuto Takahashi, Takuto Nosaka, Yosuke Murata, Ryotaro Sugata, Yu Akazawa, Tomoko Tanaka, Tatsushi Naito, Hidetaka Matsuda, Masahiro Ohani, Hiroyuki Suto, Yasunari Nakamoto

**Affiliations:** ^1^ Second Department of Internal Medicine, Faculty of Medical Sciences University of Fukui Fukui Japan; ^2^ Department of Gastroenterology Fukui General Hospital Fukui Japan; ^3^ Department of Gastroenterology Fukui Katsuyama General Hospital Fukui Japan

**Keywords:** aspirin, clopidogrel, endoscopic submucosal dissection, platelet aggregation inhibitors, stomach ulcer

## Abstract

**Objectives:**

The causes of drug‐induced gastroduodenal injuries primarily include antithrombotic drugs, including low‐dose aspirin, non‐steroidal anti‐inflammatory drugs, and corticosteroids. These drugs are suspected to affect gastric ulcer healing after endoscopic submucosal dissection (ESD). This study aimed to investigate the effects of these drugs on post‐ESD ulcer healing.

**Methods:**

This study included 170 lesions (149 patients) who had undergone ESD for early gastric cancer. Post‐ESD ulcers were endoscopically measured on the 1st, 28th, and 56th days after ESD and were analyzed to identify potential risk factors for delayed ulcer healing among patients taking antiplatelet drugs, anticoagulant drugs, non‐steroidal anti‐inflammatory drugs, corticosteroids, and no drugs.

**Results:**

Multivariate analysis for the frequency of scarring on the 56^th^ day after ESD showed that antiplatelet drugs (odds ratio [OR], 3.905; *p* = 0.017), ulcer size of ≥40 mm on the first day (OR, 4.903; *p* = 0.006), hemoglobin A1c ≥6.5% (OR 7.659, *p* = 0.012), and age of ≥75 (OR, 5.227; *p* = 0.007) were independent risk factors of delayed ulcer healing. Anticoagulant drugs, non‐steroidal anti‐inflammatory drugs, and corticosteroids were not significant factors. Among antiplatelet drugs, the ulcer reduction ratio for clopidogrel on the 28th day after ESD was 84.8%, which was significantly lower than the ulcer reduction ratio of 92.8% for no drug (*p* < 0.05).

**Conclusions:**

As antiplatelet drugs, particularly clopidogrel, may delay gastric ulcer healing after ESD, careful endoscopic follow‐up and drug therapy are suggested for patients taking these drugs.

## INTRODUCTION

Endoscopic submucosal dissection (ESD) for early‐stage gastric cancer and adenoma has become very common in recent years.^1^ In Japan, the Japan Gastroenterological Endoscopy Society has developed clinical guidelines for gastric ESD that describe the indications, treatment methods, and complications.^2^ Post‐ESD bleeding is the most common complication, and acid secretion inhibitors, including proton pump inhibitors, are recommended for its prevention. Antithrombotic drugs, tumor size, tumor localization, and chronic kidney disease treated with hemodiafiltration have been reported as risk factors for post‐ESD bleeding.^3^

Low‐dose aspirin (LDA), an antiplatelet drug, causes gastrointestinal mucosal injury and can be a risk factor for post‐ESD bleeding. Nonsteroidal anti‐inflammatory drugs (NSAIDs), including LDA, cause gastric mucosal injury, which is categorized as the inhibition of prostaglandin (PG) synthesis through cyclooxygenase (COX) inhibition and direct mucosal injury.^4^, ^5^, ^6^ PG deficiency in the gastric mucosa, which is mediated by COX inhibition, leads to decreased bicarbonate secretion and mucosal blood flow, resulting in mucosal injury. Aspirin also causes tight junction injury in the gastric epithelium, which has been shown to directly damage the gastric mucosa. Corticosteroids can cause gastrointestinal mucosal injuries. However, recent reports have indicated that corticosteroid administration alone does not increase the risk of peptic ulcer disease.^7^, ^8^

It is assumed, but not clear, that the various drugs that cause gastric mucosal injury may affect not only post‐ESD bleeding but also ulcer healing after ESD. Since an increasing number of patients have recently undergone ESD while taking these drugs, consideration of ulcer healing after ESD is also clinically important in determining the duration of treatment, including the administration of acid secretion inhibitors. By clarifying the clinical background of delayed ulcer healing, it will be possible to determine the treatment period appropriate for each individual case. Inadequate treatment of post‐ESD ulcers may lead to complications such as post‐ESD bleeding. This study investigated the influence of drugs causing gastrointestinal injury, including antithrombotic agents, NSAIDs, and corticosteroids, on ulcer healing after ESD in patients with early‐stage gastric cancer.

## METHODS

### Study design and patients

**FIGURE 1:**
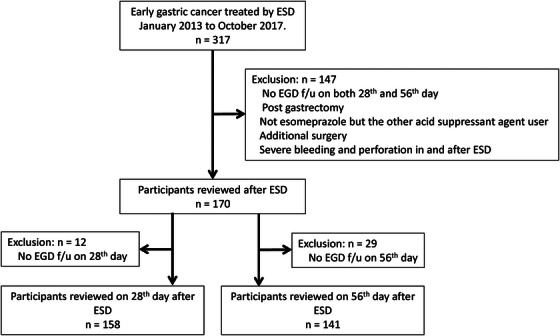
The study was ultimately conducted on 170 lesions (149 patients) treated with ESD for early gastric cancer between January 2013 and October 2017. Of the 317 consecutive cases of early gastric cancer lesions diagnosed via endoscopy and histopathology, 147 were excluded based on the exclusion criteria. ESD, endoscopic submucosal dissection; EGD, esophagogastroduodenoscopy.

This study included 170 lesions (149 patients) treated with ESD for early gastric cancer between January 2013 and October 2017 at the University of Fukui Hospital. This study was conducted with informed consent in the form of opt‐out on the website and was approved by the institutional review board of our facility (approval number: 20150129). Moreover, this observational study was a retrospective analysis. Of the 317 consecutive early gastric cancer lesions diagnosed using endoscopy and histopathology, 147 were excluded based on the following exclusion criteria: no endoscopy follow‐up, post‐gastrectomy, not esomeprazole but other acid suppressant agent users, additional surgery, severe bleeding, and perforation in and after ESD. Severe bleeding was defined as post‐ESD bleeding, including overt hematemesis and melena or a decrease in hemoglobin of 2 g/dL or more. Those lesions were excluded because bleeding made it impossible to measure the ulcer on the day after ESD or because it required an extended period of antithrombotic withdrawal. Of the 170 lesions targeted, 158 were observed on day 28 and 141 were observed on day 56 after ESD (Figure 1). We observed post‐ESD ulcers in these patients to investigate the factors that may influence ulcer healing. Antiplatelet drugs (LDA, clopidogrel, cilostazol, dipyridamole, and ethyl icosapentate [EPA]), anticoagulants (warfarin and direct oral anticoagulants [DOACs]), NSAIDs, and corticosteroids are deemed as drugs that may affect ulcer healing. The patients taking drugs (antiplatelet drugs, anticoagulants, NSAIDs, and corticosteroids) were defined as those who had been administered the medication before ESD and continued to take the medication after ESD. For antithrombotic drugs, LDA and clopidogrel were withdrawn from 5 days prior to ESD; cilostazol, dipyridamole, and EPA were withdrawn on the day of ESD; warfarin was replaced with heparin or withdrawn from 5 days prior to ESD; DOAC was withdrawn on the day of ESD. The withdrawn drugs were resumed from day 2 post‐ESD; cases with prolonged withdrawal periods due to post‐ESD bleeding were excluded. The number of patients taking these drugs was 46 (LDA, 28; clopidogrel, 14; cilostazol, 5; dipyridamole, 1; and EPA, 1), 14 (warfarin, 10; and DOAC, 4), 6, and 3, respectively. Three patients were administered a combination of LDA and clopidogrel.

### ESD for gastric tumor

We performed ESD for gastric lesions using either a conventional single‐channel endoscope (GIF‐Q260J; Olympus) or a multi‐bending two‐channel endoscope (GIF‐2TQ260M; Olympus). An IT knife 2 (KD‐611L; Olympus) and/or a Dual Knife (KD‐655L; Olympus) was used, and an electrosurgical current was applied using an electrosurgical generator (VIO300D; ERBE Elektromedizin GmbH). In some cases, the visible vessels were heat‐coagulated using hemostatic forceps (Coagrasper, FD‐410LR; Olympus).

### Endoscopic follow‐up schedule and measurement of ulcers

**FIGURE 2:**
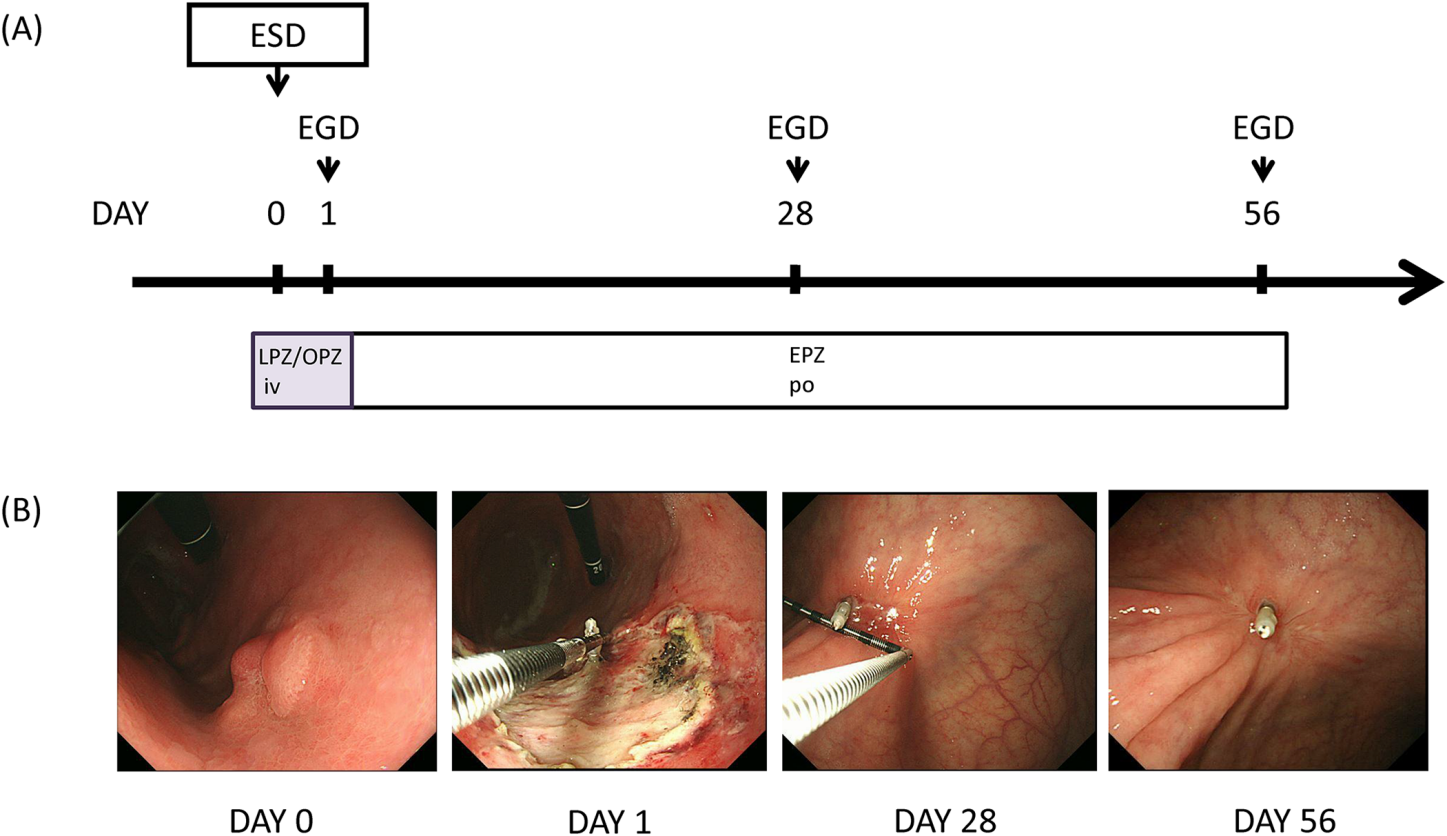
(a) ESD was performed on day 0. Omeprazole or lansoprazole injections were administered intravenously on days 0–1. The oral administration of esomeprazole continued on the 56th day after ESD. (b) The length of the major and minor axes of the post‐ESD ulcers was measured, and the ulcerated areas were calculated on the 1st, 28th, and 56th days after ESD. ESD, endoscopic submucosal dissection; EGD, esophagogastroduodenoscopy; OPZ, omeprazole; LPZ, lansoprazole; EPZ, esomeprazole.

Either omeprazole or lansoprazole was administered intravenously on days 0–1. Subsequently, the oral administration of esomeprazole 20 mg was continued until the 56th day after ESD. Endoscopic follow‐ups were performed on days 1, 28, and 56 post‐ESD (Figure 2). The frequency of scarring on the 28th and 56th days after ESD was evaluated to identify the potential risk factors for delayed ulcer healing. The lengths of the major and minor axes of the post‐ESD artificial ulcers were measured using a straight or bent type of endoscopic measure (M1‐2K and M2‐3U; Olympus) during endoscopy on days 1, 28, and 56 post‐ESD.

### Statistical analysis

Patient characteristics, including age, pre‐ESD laboratory data, tumor size, specimen size, procedure time, length of ulcer, artificial ulcer area, and ulcer reduction ratio, were expressed as mean ± standard deviation (SD).

Artificial ulcer areas approximating an oval shape and ulcer reduction ratio were calculated on day 28 after ESD as follows:

Ulcer area (mm^2^) = a/2 × b/2 × π, a and b represent the lengths of the major and minor axes of the ulcer.

Ulcer reduction ratio (%): (1 − ulcer area on day 28/ulcer area on day 1) × 100.

Pearson's chi‐square test, Fisher's exact test, and Mann–Whitney U test were used to compare qualitative variables. Logistic regression analysis was used for multivariate analysis. Statistical significance was set at *p* < 0.05. All statistical analyses were performed using SPSS Statistics software version 25 (SPSS Inc.; IBM Japan).

## RESULTS

### Patient characteristics

Overall, 170 early gastric cancer lesions (149 patients) treated with ESD were investigated. The mean age was 73.0 ± 9.3 (mean ± SD) and the male proportion was 70.6% (Table 1). Thirteen patients had severe co‐morbidities (7.6%). Severe comorbidities were defined as cirrhosis with portal hypertension and impaired cardiac function (NYHA ≥ II), based on the Charlson Comorbidity Index (CCI), and as severe renal dysfunction (estimated glomerular filtration rate [eGFR] < 30 mL/min/1.73 m2) based on the Clinical Practice Guidebook for diagnosis and treatment of chronic kidney disease 2012.^9^ Moreover, 15 patients (hemoglobin A1c [HbA1c] ≥6.5 %) had diabetes mellitus (8.8%). The number of patients taking drugs that may cause gastric mucosal damage, including antiplatelet drugs, anticoagulant drugs, NSAIDs, and corticosteroids, was 46 (27.1%), 14 (8.2%), six (3.5%), and three (1.8%), respectively. Smoking (Brinkman Index ≥400) at the time of ESD was found in 84 patients (49.4%). Regarding *Helicobacter pylori* infection status, 91 lesions (53.5%) were currently infected, 77 lesions (45.3%) were previously infected, and two lesions (1.2%) were uninfected. In atrophic gastritis, 149 lesions (87.6%) had severe atrophy (O‐2, O‐3) according to the Kimura‐Takemoto classification, 19 lesions (11.2%) had moderate atrophy (C‐3, O‐1), and two lesions (1.2%) had mild atrophy or no atrophy (C‐0–C‐2). Serum albumin levels and eGFR were investigated as pre‐ESD laboratory data.

**TABLE 1:** Participant characteristics.

	Overall (*n* = 170)
Age, mean ± SD, years	73.0 ± 9.3
Sex (male), *n* (%)	120 (70.6)
Severe co‐morbidity (CHF, CKD, LC), *n* (%)	13 (7.6)
Diabetes mellitus (HbA1c ≥ 6.5 %), *n* (%)	15 (8.8)
Antiplatelet drugs, *n* (%)	46 (27.1)
Anticoagulant drugs, *n* (%)	14 (8.2)
NSAIDs, *n* (%)	6 (3.5)
Corticosteroid, *n* (%)	3 (1.8)
Smoking, *n* (%)	84 (49.4)
*H. pylori* infection status	
Current *H. pylori* infection, *n* (%)	91 (53.5)
Previous *H. pylori* infection, *n* (%)	77 (45.3)
No *H. pylori* infection, *n* (%)	2 (1.2)
Atrophic gastritis status	
Severe atrophic gastritis, *n* (%)	149 (87.6)
Moderate atrophic gastritis, *n* (%)	19 (11.2)
Mild atrophic gastritis, *n* (%)	2 (1.2)
Pre‐ESD laboratory data	
Albumin, mean ± SD, g/dL	4.1 ± 0.4
eGFR, mean ± SD, mL/min/1.73m^2^	61.3 ± 18.6
Tumor‐related characteristics	
Tumor size, mean ± SD, mm	14.4 ± 9.5
Tumor location, *n* (%)	
Upper	21 (12.4)
Middle	87 (51.2)
Lower	62 (36.5)
ESD‐related characteristics	
En bloc resection, *n* (%)	166 (97.6)
Specimen size, mean ± SD, mm	33.2 ± 11.1
Procedure time, mean ± SD, min	86.5 ± 56.1
Day 1 long diameter of ulcer, mean ± SD, mm	33.7 ± 12.0

Abbreviations: CHF, congestive heart failure; CKD, chronic kidney disease; eGFR, estimated glomerular filtration rate; ESD, endoscopic submucosal dissection; H., helicobacter; HbA1c, hemoglobin A1c; LC, liver cirrhosis; NSAID, nonsteroidal anti‐inflammatory drug; SD, standard deviation.

The mean size of the resected tumor by ESD was 14.4 ± 9.5 mm, and the frequency of upper, middle, and lower thirds in tumor location was 12.4%, 51.2%, and 36.5%, respectively.

The mean size of the specimen was 33.2 ± 11.1 mm, the mean procedure time was 86.5 ± 56.1 min, and the frequency of en bloc resection was 97.6%. The long diameter of the ulcer on day 1 was 33.7 ± 12.0 mm.

### Scarring frequency of artificial ulcers after ESD

The results of univariate analysis of scarring frequency on the 28th and 56th days after ESD are shown in Table 2. Regarding scarring frequency, on the 28th day after ESD, the ulcer scarring rate was significantly lower in ulcers with a diameter of ≥40 mm on the first day than in those with a diameter of < 40 mm. Regarding scarring frequency on the 56^th^ day after ESD, the ulcer scarring rate was significantly lower in cases of antiplatelet agents, ulcer size of ≥40 mm on the first day, HbA1c ≥6.5%, and age ≥75 years.

**TABLE 2:** Univariate analysis of scarring frequency.

	Frequency of scarring, *n* (%) on the 28th day after ESD	*p*‐value	Frequency of scarring, *n* (%) on the 56th day after ESD	*p*‐value
Age, years		0.062		0.013^*^
≥75	5/68 (7.4)		46/60 (76.7)	
≤74	16/90 (17.8)		75/81 (92.6)	
Sex,		0.313		1.000
Male	12/108 (11.1)		85/99 (85.9)	
Female	9/50 (18.0)		36/42 (85.7)	
Severe co‐morbidity (CHF, CKD, LC)		0.662		0.601
Yes	2/12 (16.7)		8/8 (100)	
No	19/146 (13.0)		113/133 (85.0)	
HbA1c, %		0.223		0.006^*^
≥6.5	0/15 (0)		5/10 (50.0)	
< 6.5	21/143 (14.7)		116/131 (88.5)	
Antiplatelet drugs		0.194		0.012^*^
Yes	3/43 (6.9)		26/36 (72.2)	
No	18/115 (15.7)		95/105 (90.5)	
Anticoagulant drugs		0.370		0.656
Yes	0/12 (0)		9/11 (81.8)	
No	21/146 (14.4)		112/130 (86.2)	
NSAIDs		0.581		0.202
Yes	1/6 (16.7)		4/6 (66.7)	
No	20/152 (13.2)		117/135 (86.7)	
Corticosteroid		1.000		1.000
Yes	0/2 (0)		3/3 (100.0)	
No	21/156 (13.5)		118/138 (85.5)	
Smoking		0.484		0.476
Yes	8/74 (10.8)		60/68 (88.2)	
No	13/84 (15.5)		61/73 (83.6)	
*H. pylori* current infection		0.347		0.810
Yes	14/87 (16.1)		67/79 (84.8)	
No	7/71 (9.9)		54/62 (87.1)	
Severe atrophic gastritis		1.000		1.000
Yes	19/138 (13.8)		109/127 (85.8)	
No	2/20 (10.0)		12/14 (85.7)	
Albumin, g/dL		0.642		1.000
≤3.5	2/11 (18.2)		9/10 (90.0)	
> 3.5	19/147 (12.9)		112/131 (85.5)	
eGFR, mL/min/1.73m^2^		0.488		0.461
< 60	11/71 (15.7)		47/57 (82.5)	
≥60	10/87 (11.5)		74/84 (88.1)	
Tumor location		0.853		0.335
Upper	2/17 (11.8)		14/16 (87.5)	
Middle	10/82 (12.2)		67/75 (89.3)	
Lower	9/59 (15.2)		40/50 (80.0)	
DAY1 long diameter of ulcer, mm		0.004^*^		0.004^*^
≥40	1/50 (2.0)		32/44 (76.2)	
< 40	20/108 (18.5)		89/97 (91.8)	

Abbreviations: CHF, congestive heart failure; CKD, chronic kidney disease; eGFR, estimated glomerular filtration rate; ESD, endoscopic submucosal dissection; H., helicobacter; HbA1c, hemoglobin A1c; LC, liver cirrhosis; NSAID, nonsteroidal anti‐inflammatory drug; SD, standard deviation.

* *p* < 0.05.

Multivariate analysis of scarring frequency on the 56th day after ESD showed that antiplatelet drugs (odds ratio [OR], 3.905; *p* = 0.017), ulcer size of ≥40 mm on the first day (OR, 4.903; *p* = 0.006), HbA1c ≥6.5% (OR, 7.659; *p* = 0.012), and age of ≥75 (OR, 5.227; *p* = 0.007) were revealed to be independent risk factors of delayed ulcer healing. These four factors may be involved in the delayed healing of post‐ESD ulcers. Anticoagulants, NSAIDs, and corticosteroids were not risk factors (Table 3).

**TABLE 3:** Multivariate analysis of scarring frequency on the 56th day after endoscopic submucosal dissection.

	OR	95% CI	*p*‐value
Age ≥75 years	5.227	1.560–17.508	0.007^*^
HbA1c ≥6.5%	7.659	1.566–37.459	0.012^*^
Antiplatelet drugs	3.905	1.272–11.988	0.017^*^
Day 1 long diameter of ulcer ≥40 mm	4.903	1.566–15.352	0.006*

Abbreviations: CI, confidence interval; ESD, endoscopic submucosal dissection; HbA1c, hemoglobin A1c; OR, odds ratio.

* *p* < 0.05.

### Ulcer reduction ratios of the artificial ulcer area in the antiplatelet drug group

Thereafter, we compared the ulcer reduction ratios on the 28th day after ESD in 43 patients in the antiplatelet drug group and 98 patients in the no‐drug group. Regarding the ulcer reduction ratio on the 56th day after ESD, 121 of 141 lesions had scarring of post‐ESD ulcers (100% ulcer reduction ratio) and there were no significant differences in the ulcer reduction ratios of various clinical characteristics, including the use of antiplatelet drugs. The mean age of the antiplatelet drug group was 76.5 ± 6.6 years, which was significantly older than that of the no drugs group at 71.3 ± 9.7 years (Table 4). Patients in the antiplatelet drug group had significantly higher rates of severe comorbidities, including chronic heart failure, chronic kidney disease, and liver cirrhosis. Pre‐ESD laboratory data showed significant differences in serum albumin levels and eGFR between both groups. However, there were no differences in diabetes mellitus (HbA1c ≥6.5%) and day 1 long diameter.

**TABLE 4:** Characteristics of the antiplatelet drug and no‐drug groups.

	Antiplatelet drugs (*n* = 43)	No drugs (*n* = 98)	*p*‐value
Age, mean ± SD, years	76.5 ± 6.6	71.3 ± 9.7	0.002^*^
Sex (male), *n* (%)	34 (79.1)	63 (64.3)	0.114
Severe co‐morbidity (CHF, CKD, and LC), *n* (%)	7 (16.3)	5 (5.1)	0.045^*^
Diabetes mellitus (HbA1c ≧ 6.5%), *n* (%)	5 (11.6)	7 (7.1)	0.512
Pre‐ESD laboratory data			
Albumin, mean ± SD, g/dL	4.0 ± 0.3	4.1 ± 0.4	0.019^*^
eGFR, mean ± SD, mL/min/1.73m^2^	52.4 ± 17.9	65.0 ± 17.5	<0.001^*^
Smoking, *n* (%)	20 (46.5)	44 (44.9)	1.000
*H. pylori* current infection, *n* (%)	25 (58.1)	54 (55.1)	0.852
Severe atrophic gastritis, *n* (%)	38 (88.4)	83 (84.7)	0.794
Tumor‐related characteristics			
Tumor size, mean ± SD, mm	12.7 ± 7.2	14.8 ± 10.2	0.426
Tumor location, *n* (%)			
Upper	5 (11.6)	12 (12.2)	1.000
Middle	18 (41.9)	54 (55.1)	0.200
Lower	20 (46.5)	32 (32.7)	0.132
ESD‐related characteristics			
En bloc resection, *n* (%)	42 (97.7)	95 (96.9)	1.000
Specimen size, mean ± SD, mm	30.5 ± 7.9	33.6 ± 11.8	0.246
Procedure time, mean ± SD, min	73.6 ± 37.2	90.6 ± 60.7	0.189
Day 1 long diameter of ulcer, mean ± SD, mm	31.3 ± 10.5	34.0 ± 12.3	0.282

Abbreviations: CHF chronic heart failure; CKD, chronic kidney disease; ESD, endoscopic submucosal dissection; LC liver cirrhosis; SD, standard deviation.

* *p* < 0.05.

The ulcer reduction ratio in the antiplatelet drug group on the 28th day after ESD was 89.5% ± 10.4%, which was significantly lower than that of the no‐drug group at 92.8% ± 8.62% (Table 5). In the antiplatelet drug group, the ulcer area reduction ratios for LDA, clopidogrel, and cilostazol were 89.9% (LDA), 84.8% (clopidogrel), and 97.4% (cilostazol), respectively. The ulcer area reduction ratios of clopidogrel were significantly lower than those of the no‐drug group (*p* < 0.05), but no statistical difference was observed in LDA compared with that of the no‐drug group (*p* = 0.153). This suggests that antiplatelet drugs, particularly clopidogrel, delay ulcer healing after gastric ESD.

**TABLE 5:** Ulcer reduction ratio on the 28th day after endoscopic submucosal dissection (ESD).

	Reduction ratio, mean ± SD %	*p*‐value	
Antiplatelet drugs versus no drugs,	89.5 ± 10.4	92.8 ± 8.2	0.029^*^
Low‐dose aspirin versus no drugs, *n* = 26	89.9 ± 11.2	92.8 ± 8.2	0.153
Clopidogrel versus no drugs, *n* = 13	84.8 ± 9.9	92.8 ± 8.2	0.003^*^
Cilostazol versus no drugs, *n* = 5	97.4 ± 2.6	92.8 ± 8.2	0.271

Abbreviation: SD, standard deviation.

* *p* < 0.05.

## DISCUSSION

This study suggests that the healing of post‐ESD ulcers may be delayed in patients receiving antiplatelet drugs. The ulcer scarring frequency on the 56th day after ESD was significantly lower in the antiplatelet group than that in the no‐drug group, and the ulcer reduction ratio on the 28th day after ESD was significantly lower in the antiplatelet drug group, especially clopidogrel than that in the no‐drug group. These results are important when considering the duration of treatment, including the administration of acid secretion inhibitors, including proton pump inhibitors, in ulcer healing after gastric ESD.

As mentioned previously, gastrointestinal mucosal injury caused by antiplatelet drugs has been reported with low‐dose aspirin, which may have important implications for ulcer healing after gastric ESD. Although the single administration of clopidogrel has no effect on the gastrointestinal mucosa and does not increase the risk of gastrointestinal bleeding,^10^ Ng et al. reported that clopidogrel use among 70 patients with a history of peptic ulcers and aspirin‐induced upper gastrointestinal complications resulted in a significantly higher rate of gastrointestinal bleeding in those with upper gastrointestinal bleeding than in those without.^11^ This suggests that even clopidogrel alone may increase the risk of gastrointestinal bleeding in patients at high risk of bleeding, including those with a history of upper gastrointestinal bleeding. After gastric ESD, a combination of aspirin and clopidogrel has been reported to increase the risk of postoperative bleeding,^12^, ^13^ while aspirin monotherapy does not increase the risk of postoperative bleeding.^13^ However, bleeding and delayed ulcer healing after gastric ESD may be caused by different pathologies; notably, delayed ulcer healing was observed in the clopidogrel group, which is considered to have no mucosal injury effect.

Among antithrombotic drugs, ticagrelor and clopidogrel have been shown in animal studies to impair ulcer healing rather than mucosal injury owing to their angiogenesis‐inhibitory effects.^14^, ^15^ Clopidogrel has been shown to decrease the expression of epidermal growth factor receptor and phosphorylated extracellular signal‐regulated kinase, inhibit proliferation of gastric mucosal epithelial cells,^16^ and suppress angiogenesis via the vascular endothelial growth factor receptor signaling pathway.^17^, ^18^ Therefore, clopidogrel may delay the healing of erosions and small ulcers and promote bleeding due to its angiogenesis‐inhibiting characteristics.^19^ These findings suggest that clopidogrel delays ulcer healing after gastric ESD. It is important to mention the interaction between clopidogrel and proton pump inhibitors (PPIs). Clopidogrel and PPIs, especially omeprazole, share the drug‐metabolizing enzyme CYP2C19, and therefore PPIs inhibit the conversion of clopidogrel to its active metabolites. This reduces the platelet aggregation inhibitory effect, which has been reported to be associated with an increase in cardiovascular events.^20^ However, in the Japanese population, no significant association was found between the concomitant use of PPIs and clopidogrel and cardiovascular events.^21^, ^22^ Esomeprazole may inhibit the activation of clopidogrel via CYP2C19, potentially reducing its antiplatelet effect, which may reduce its effect in delaying ulcer healing after ESD.

Factors that promote gastric ulcer healing after ESD include the administration of acid secretion inhibitors and gastric mucosal protectants, including PPIs, which are useful in preventing post‐ESD bleeding.^23^, ^24^ Moreover, vonoprazan was as effective as or more effective than PPIs for healing post‐ESD ulcers.^25^, ^26^ Gastric pH may influence the healing of post‐ESD ulcers. Other factors reported to delay post‐ESD ulcer healing include upper or middle stomach lesions,^27^ large ulcer sizes after ESD, and diabetes, similar to this study.^28^, ^29^ In addition, the results of this study showed that ulcer healing after ESD was delayed in patients aged 75 years or older. The aging gastric mucosa has reduced mucosal defense and is more susceptible to injury from a variety of toxic substances, including aspirin, other NSAIDs, and ethanol, which can delay or impair the healing of the injury. The mechanisms underlying these abnormalities in the aged gastric mucosa include reduced mucosal blood flow leading to hypoxia.^30^

A limitation is that this study was completed and the data was analyzed in 2017. However, this does not mean that the study is outdated, as endoscopic treatment techniques for early gastric cancer and the clinical background of the patients to be treated have not changed. In conclusion, the administration of antiplatelet drugs, especially clopidogrel, may delay gastric ulcer healing after ESD. These results suggest that these drugs not only cause bleeding but may also affect mucosal healing following endoscopic treatment.

## CONFLICT OF INTEREST STATEMENT

None.

## ETHICS STATEMENT

Approval of the research protocol by an Institutional Reviewer Board: This study was approved by the institutional review board of our facility (approval number: 20150129).

## PATIENT CONSENT STATEMENT

This study was conducted with informed consent in the form of opt‐out on the website.

## CLINICAL TRIAL REGISTRATION

N/A.

## Data Availability

Raw data was generated at the University of Fukui. Derived data supporting the findings of this study are available from the corresponding author Yasunari Nakamoto on request.

## References

[1] Fujishiro M . Endoscopic submucosal dissection for stomach neoplasms. World J Gastroenterol 2006; 12: 5108–5112. 16937520 10.3748/wjg.v12.i32.5108 PMC4088006

[2] Ono H , Yao K , Fujishiro M *et al*. Guidelines for endoscopic submucosal dissection and endoscopic mucosal resection for early gastric cancer. Dig Endosc 2016; 28: 3–15. 26234303 10.1111/den.12518

[3] Hatta W , Tsuji Y , Yoshio T *et al*. Prediction model of bleeding after endoscopic submucosal dissection for early gastric cancer: BEST‐J score. Gut 2021; 70: 476–484. 32499390 10.1136/gutjnl-2019-319926 PMC7873424

[4] Schoen RT , Vender RJ . Mechanisms of nonsteroidal anti‐inflammatory drug induced gastric damage. Am J Med 1989; 86: 449–58. 2648824 10.1016/0002-9343(89)90344-6

[5] Meyer RA , McGinley D , Posalaky Z . The effects of 16, 16‐dimethyl prostaglandin E2+aspirin on the canine gastric mucosal barrier. Virchows Arch A Pathol Anat Histopathol 1987; 412: 119–125. 3122412 10.1007/BF00716183

[6] Wallace JL , McKnight W , Reuter BK , Vergnolle N . NSAID‐induced gastric damage in rats: Requirement for inhibition of both cyclooxygenase 1 and 2. Gastroenterology 2000; 119: 706–714. 10982765 10.1053/gast.2000.16510

[7] Conn HO , Poynard T . Corticosteroids and peptic ulcer: Meta‐analysis of adverse events during steroid therapy. J Intern Med 1994; 236: 619–632. 7989897 10.1111/j.1365-2796.1994.tb00855.x

[8] Piper JM , Ray WA , Daugherty JR , Griffin MR . Corticosteroid use and peptic ulcer disease: Role of nonsteroidal anti‐inflammatory drugs. Ann Intern Med 1991; 114: 735–740. 2012355 10.7326/0003-4819-114-9-735

[9] Japan nephrology society . Clinical practice guidebook for diagnosis and treatment of chronic kidney disease 2012. Nihon Jinzo Gakkai Shi 2012; 54: 1034–1191. 23387281

[10] Hallas J , Dall M , Andries A *et al*. Use of single and combined antithrombotic therapy and risk of serious upper gastrointestinal bleeding: Population based case‐control study. BMJ 2006; 333: 726. 16984924 10.1136/bmj.38947.697558.AE PMC1592384

[11] Ng FH , Wong SY , Chang CM *et al*. High incidence of clopidogrel‐associated gastrointestinal bleeding in patients with previous peptic ulcer disease. Aliment Pharmacol Ther 2003; 18: 443–449. 12940930 10.1046/j.1365-2036.2003.01693.x

[12] Takeuchi T , Ota K , Harada S *et al*. The postoperative bleeding rate and its risk factors in patients on antithrombotic therapy who undergo gastric endoscopic submucosal dissection. BMC Gastroenterol 2013; 13: 136. 24010587 10.1186/1471-230X-13-136 PMC3844538

[13] Tounou S , Morita Y , Hosono T . Continuous aspirin use dose not increase post‐endoscopic dissection bleeding risk for gastric neoplasms in patients on antiplatelet therapy. Endosc Int Open 2015; 3: E31–38. 26134769 10.1055/s-0034-1390764 PMC4423265

[14] Ma L , Elliott SN , Cirino G , Buret A , Ignarro LJ , Wallace JL . Platelets nodulate gastric ulcer healing: Role of endostatin and vascular endothelial growth factor release. Proc Natl Acad Sci U S A 2001; 98: 6470–6475. 11353854 10.1073/pnas.111150798 PMC33492

[15] Li JJ , Wu XY , Chen JL *et al*. Antiplatelet drug ticagrelor delays gastric ulcer healing in rats. Exp Ther Med 2017; 14: 3774–3779. 29042978 10.3892/etm.2017.4955 PMC5639414

[16] Luo JC , Huo TI , Hou MC *et al*. Clopidogrel delays gastric ulcer healing in rats. Eur J Pharmacol 2012; 695: 112–119. 22975710 10.1016/j.ejphar.2012.07.054

[17] Luo JC , Peng YL , Chen TS *et al*. Clopidogrel inhibits angiogenesis of gastric ulcer healing via downregulation of vascular endothelial growth factor receptor 2. J Formos Med Assoc 2016; 115: 764–72. 26315480 10.1016/j.jfma.2015.07.022

[18] Wu H , Wei M , Li N *et al*. Clopidogrel‐induced gastric injury in rats is attenuated by stable gastric pentadecapeptide BPC 157. Drug Des Devel Ther 2020; 14: 5599–5610. 10.2147/DDDT.S284163 PMC7763470 33376304

[19] Cryer B . Reducing the risks of gastrointestinal bleeding with antiplatelet therapies. N Engl J Med 2005; 352: 287–289. 15659730 10.1056/NEJMe048330

[20] Ho PM , Maddox TM , Wang L *et al*. Risk of adverse outcomes associated with concomitant use of clopidogrel and proton pump inhibitors following acute coronary syndrome. JAMA 2009; 301: 937–944. 19258584 10.1001/jama.2009.261

[21] Aihara H , Sato A , Takeyasu N *et al*. Effect of individual proton pump inhibitors on cardiovascular events in patients treated with clopidogrel following coronary stenting: Results from the Ibaraki Cardiac Assessment Study Registry. Catheter Cardiovasc Interv 2012; 80: 556–563. 22234956 10.1002/ccd.23327

[22] Chitose T , Hokimoto S , Oshima S *et al*. Clinical outcomes following coronary stenting in Japanese patients treated with and without proton pump inhibitor. Circ J 2012; 76: 71–78. 22130313 10.1253/circj.cj-11-0699

[23] Uedo N , Takeuchi Y , Yamada T *et al*. Effect of a proton pump inhibitor or an H2‐receptor antagonist on prevention of bleeding from ulcer after endoscopic submucosal dissection of early gastric cancer: A prospective randomized controlled trial. Am J Gastroenterol 2007; 102: 1610–6. 17403076 10.1111/j.1572-0241.2007.01197.x

[24] Yang Z , Wu Q , Liu Z , Wu K , Fan D . Proton pump inhibitors versus histamine‐2‐receptor antagonists for the management of iatrogenic gastric ulcer after endoscopic mucosal resection or endoscopic submucosal dissection: A meta‐analysis of randomized trials. Digestion 2011; 84: 315–320. 22075541 10.1159/000331138

[25] Maruoka D , Arai M , Kasamatsu S *et al*. Vonoprazan is superior to proton pump inhibitors in healing artificial ulcers of the stomach post‐endoscopic submucosal dissection: A propensity score‐matching analysis. Dig Endosc 2017; 29: 57–64. 27492962 10.1111/den.12705

[26] Ichida T , Ueyama S , Eto T , Kusano F , Sakai Y . Randomized controlled trial comparing the effects of vonoprazan plus rebamipide and esomeprazole plus rebamipide on gastric ulcer healing induced by endoscopic submucosal dissection. Intern Med 2019; 58: 159–166. 30210115 10.2169/internalmedicine.1146-18 PMC6378166

[27] Yoshizawa Y , Sugimoto M , Sato Y *et al*. Factors associated with healing of artificial ulcer after endoscopic submucosal dissection with reference to Helicobacter pylori infection, CYP2C19 genotype, and tumor location: Multicenter randomized trial. Dig Endosc 2016; 28: 162–172. 26331711 10.1111/den.12544

[28] Lim JH , Kim SG , Choi J , Im JP , Kim JS , Jung HC . Risk factors of delayed ulcer healing after gastric endoscopic submucosal dissection. Surg Endosc 2015; 29: 3666–3673. 25740642 10.1007/s00464-015-4123-z

[29] Shimozato A , Sasaki M , Ogasawara N *et al*. Risk factors for delayed ulcer healing after endoscopic submucosal dissection of gastric neoplasms. J Gastrointestin Liver Dis 2017; 26: 363–368. 29253050 10.15403/jgld.2014.1121.264.kas

[30] Tarnawski AS , Ahluwalia A . Increased susceptibility of aging gastric mucosa to injury and delayed healing: Clinical implications. World J Gastroenterol 2018; 24: 4721–4727. 30479459 10.3748/wjg.v24.i42.4721 PMC6235800

